# Iron nitrosyl complexes are formed from nitrite in the human placenta

**DOI:** 10.1016/j.jbc.2022.102078

**Published:** 2022-05-26

**Authors:** George T. Mukosera, Patricia Principe, Eugenia Mata-Greenwood, Taiming Liu, Hobe Schroeder, Mana Parast, Arlin B. Blood

**Affiliations:** 1 Lawrence D Longo Center for Perinatal Biology, Loma Linda University, Loma Linda, California, USA; 2 Division of Neonatology, Department of Pediatrics, Loma Linda University School of Medicine, Loma Linda, California, USA; 3 Department of Pathology, University of California San Diego, La Jolla, California, USA

**Keywords:** placenta, preeclampsia, maternal inflammation, nitric oxide, nitrite, iron nitrosyl complexes, FeNO, DNIC, DETA, diethylenetriamine, DFO, deferoxamine, DNIC, dinitrosyl iron complex, eNOS, endothelial NOS, EPR, electron paramagnetic resonance, FeNO, iron nitrosyl complex, HbNO, nitrosyl hemoglobin, HM, heavy mitochondrial, iNOS, inducible NOS, NiR, nitrate reductase, NO, nitric oxide, NOS, nitric oxide synthase, PIH, pyridoxal isonicotinoyl hydrazone, SNO, S-nitrosothiol

## Abstract

Placental nitric oxide (NO) is critical for maintaining perfusion in the maternal-fetal-placental circulation during normal pregnancy. NO and its many metabolites are also increased in pregnancies complicated by maternal inflammation such as preeclampsia, fetal growth restriction, gestational diabetes, and bacterial infection. However, it is unclear how increased levels of NO or its metabolites affect placental function or how the placenta deals with excessive levels of NO or its metabolites. Since there is uncertainty over the direction of change in plasma levels of NO metabolites in preeclampsia, we measured the levels of these metabolites at the placental tissue level. We found that NO metabolites are increased in placentas from patients with preeclampsia compared to healthy controls. We also discovered by ozone-based chemiluminescence and electron paramagnetic resonance that nitrite is efficiently converted into iron nitrosyl complexes (FeNOs) within the human placenta and also observed the existence of endogenous FeNOs within placentas from sheep and rats. We show these nitrite-derived FeNOs are relatively short-lived, predominantly protein-bound, heme-FeNOs. The efficient formation of FeNOs from nitrite in the human placenta hints toward the importance of both nitrite and FeNOs in placental physiology or pathology. As iron nitrosylation is an important posttranslational modification that affects the activity of multiple iron-containing proteins such as those in the electron transport chain, or those involved in epigenetic regulation, we conclude that FeNOs merit increased study in pregnancy complications.

Nitric oxide (NO) is a ubiquitous gasotransmitter involved in many biological functions such as vasodilation. Endogenous NO is mainly produced from the reaction of L-arginine and O_2_, catalyzed by nitric oxide synthases (NOSs). Once produced, NO is a reactive-free radical with a short half-life (<2 s) under biological conditions. NO rapidly turns into various metabolites (NOx) including nitrite, nitrate, S-nitrosothiols (SNOs), peroxynitrite, and nitrotyrosine, in oxidative reactions that are facilitated by metalloproteins (1). Direct nitrosylation reactions with either heme or nonheme iron centers of proteins can also yield iron nitrosyl complexes (FeNOs) (2).

Within the human placenta, locally produced NO from both placental endothelial NOS (eNOS) and inducible NOS (iNOS) mediates vasodilation through the cyclic guanosine monophosphate-dependent pathway. Placental NO is critical for maintaining the increased perfusion in both maternal and the fetal circulations in the placenta during pregnancy (3). The placenta therefore encounters both NO and its metabolites, and the levels of NO and NOx are likely higher under inflammatory conditions due to overexpression of both systemic and local iNOS (4). Consumption of dietary nitrate and nitrite during pregnancy (5), which can be converted to NO under specific conditions (6), could also increase the amount of NO and NOx reaching the placenta during pregnancy. Nitrate has also been proposed as a dietary means of increasing NO bioactivity in pregnancy pathologies such as preeclampsia (7). Therefore, the placenta is exposed to NO and its derivatives through endogenous NO production under normal or inflammatory conditions as well as through dietary nitrate and nitrite.

NO and NOx species are important beyond their role in vasodilation in the placenta. NO is known to actively regulate trophoblast invasion, placental angiogenesis, and vascular development, as well as placental vascular function (3, 8). Changes in placental tissue levels of eNOS and iNOS, as well as changes in plasma levels of NO metabolites, have also been observed in pregnancy pathologies such as gestational diabetes (9, 10), preeclampsia (11, 12), and fetal growth restriction (13, 14). Although the importance of NO to placental function is clear, the molecular pathways through which NO exerts its effects on placental function are less certain. Also unclear are the mechanisms by which the placenta metabolizes NO, and whether the NO metabolites independently affect placental function. The proposed mechanisms through which NO and its metabolites affect placental function include a myriad of posttranslational modifications of proteins via nitrosation of thiol residues, nitration of tyrosine residues, and nitrosylation of protein-bound iron. These modifications lead to changes in protein activity with wide-ranging effects on cellular homeostasis that include mitochondrial function (15), cell apoptosis (16), and histone methylation and acetylation, thus affecting the epigenetic regulation of gene expression (3, 8).

Iron nitrosylation is another posttranslational modification through which NO could exert its control over protein expression and function in the placenta. In tissues other than the placenta, NO is known to bind to both heme and nonheme iron centers of proteins, resulting in the formation of heme-NO or dinitrosyl iron complexes (DNICs), which we herein refer to collectively as FeNOs (2). These complexes could act to regulate concentrations of free NO by protecting it from irreversible oxidation (17), thus increasing NO bioavailability which could regulate placental function in both normal and complicated pregnancy. Complex IV of the electron transport chain (18), mitochondrial aconitases (19), and iron regulatory protein I (20, 21) have all been demonstrated to form either heme-NO or DNICs in the presence of upregulated NO, leading to altered protein function. FeNO formation is also associated with loss of demethylation activity in the JMJC domain-containing demethylases, which perform the majority of histone demethylation, leading to an overall increase in histone methylation (22). Thus, FeNOs are a means of posttranslational modification by which NO or NOx species may affect placental function, apart from the canonical vasodilatory functions of NO. However, there are virtually no studies about the existence or function of FeNOs in the placenta in either healthy or pathological pregnancies.

We recently reported that NOx species are increased at the placental tissue level in placentas from mothers with babies who had fetal growth restriction, with or without preeclampsia. We also reported that a small pool of the NOx present endogenously in the mouse placenta is in the form of FeNOs and that the placenta responds to excess NO/NOx partially by forming FeNOs (13). Although the FeNO levels that we observed were too low to allow advanced characterization, they serve as evidence that FeNOs exist in the placenta under both physiological and pathological conditions. The sparsity of studies exploring NOx formation at the placental tissue level and the relative difficulty in measuring the FeNO species with enough sensitivity for detection at physiological levels might explain why they have not been explored in the placenta. However, our recently developed ozone-based chemiluminescence assay has allowed us to reliably detect these and other NOx species with high sensitivity (23). This assay enables characterization of the metabolism of NO and its metabolites in the placenta, knowledge that is essential for determination of the mechanisms through which NO affects placental function in both normal and complicated pregnancies.

In the present study, using human term placental explants, we characterize the placental metabolism of NO and several classes of NOx species which are typically elevated in pregnancy complications such as maternal inflammation due to infection, preeclampsia, and fetal growth restriction. We further confirm the presence of endogenous placental FeNO in both rat and sheep placental tissue, adding to the notion that FeNOs are prevalent in the placenta of many species in both health and disease. We also tested whether nitrite can serve as a source of FeNO within the placenta and the extent to which FeNO formation affects the function of aconitase and jumonji C domain-containing demethylases, two iron-containing enzymes previously proposed to bind NO.

## Results

**Figure 1:**
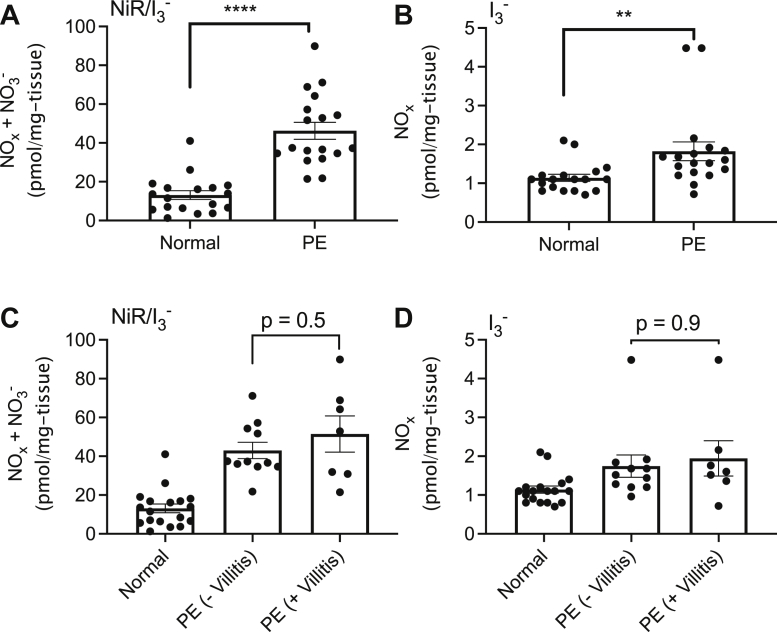
**Summary data for basal NOx metabolites in placental villous tissue from normal (n = 20) *versus* preeclamptic (PE, n = 18) pregnancies, with and without villitis, as measured by ozone-based chemiluminescence with different purge vessel reagents.** NiR/I_3_^-^ represents incubation of the placental tissue homogenates with nitrate reductase followed by injection into triiodide. NOx is defined as the sum of nitrite, nitrosothiols, and FeNOs. *A*, sum of NOx and nitrate levels in normal *versus* preeclamptic placental villous tissue. *B*, NOx differences in normal *versus* preeclamptic placental villous tissue. *C*, sum of NOx and nitrate differences in normal *versus* preeclamptic placental villous tissue with and without villitis. *D*, NOx differences in normal *versus* preeclamptic placental villous tissue with and without villitis. Preeclamptic placental tissue is associated with significantly higher levels of both NOx and nitrate than normotensive placental tissue. Villitis, however, does not result in any additional NOx increases in preeclamptic placentas. No FeNO signal was detected with the PBS/FeCN assay in both normal and preeclamptic placental tissue. *∗∗ represents p < 0.01, ∗∗∗∗ represents p < 0.0001*. NiR, nitrate reductase; FeNO, iron nitrosyl complex.

We measured the endogenous levels of NOx in placentas from uncomplicated pregnancies in comparison with placentas from pregnancies complicated with preeclampsia with or without villitis. As shown in Figure 1*A*, the total NOx (including nitrate) is significantly increased in placental tissue from preeclampsia *versus* normal pregnancy indicating that placental NO production is increased in preeclampsia. Figure 1*B* indicated the presence of significantly higher levels of bioactive NOx (nitrite + RSNO + FeNO) in placental tissue from preeclampsia *versus* normal pregnancy, as measured by the triiodide assay which does not detect nitrate. Detection of FeNO by the PBS/FeCN assay yielded no detectable signal in either the normal or preeclamptic placental tissue. Comparison of Figure 1*A* *versus* Figure 1*B* shows a 25-fold increase in NOx signal when placental tissue homogenates are incubated with nitrate reductase (NiR) before injection into triiodide, indicating nitrate to be the predominant metabolite present in preeclamptic *versus* normal placental tissue. Higher levels of nitrate are consistent with nitrate being the end-product of NO, nitrite, RSNO, and FeNO metabolism. The presence of placental villitis did not result in any additional increases in nitrate + NOx (Fig. 1*C*) or bioactive NOx alone (Fig. 1*D*) in preeclamptic placental tissue.

**Figure 2:**
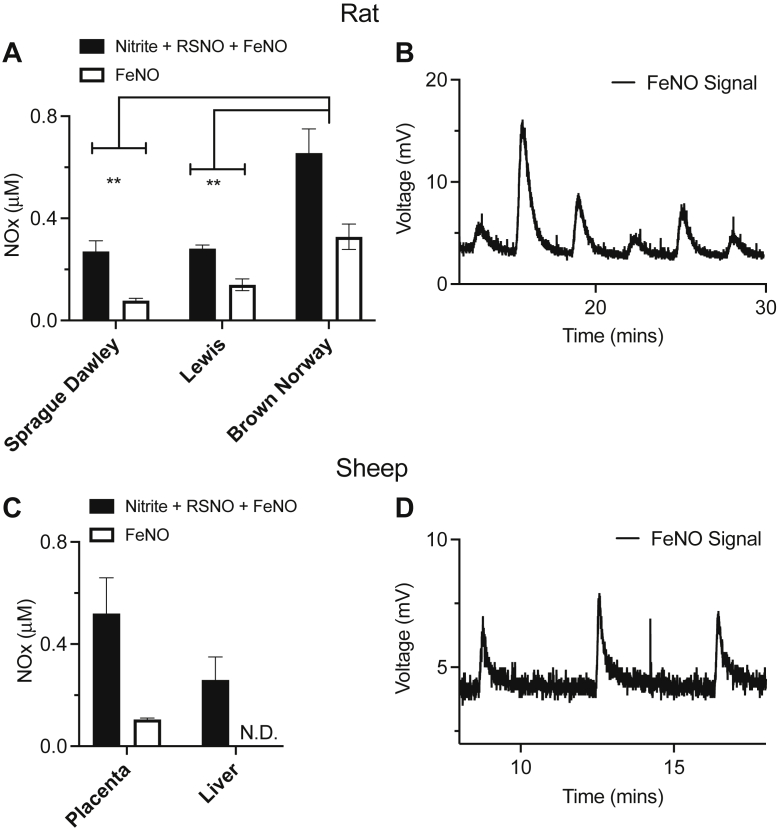
**Detection of basal levels of NOx and FeNO in placental tissue from three different rat species as well as from the sheep placenta.***A*, total bioactive NOx (nitrite + RSNO + FeNO) *versus* FeNO signal in placentas from Sprague Dawley (*n =5)*, Lewis (*n = 5*), and *Brown Norway* (*n = 5*) pregnant rats. At least 50% of the bioactive NOx signal in the rat placenta is in the form of FeNOs for Lewis and BN and 25% for SD rats. Values of both total NOx and FeNO are significantly higher in the Brown Norway rats than either the Sprague Dawley (*p* < 0.001) or the Lewis rats (*p* < 0.001). *B*, the voltage tracing of the FeNO signal from the rat placenta. *C*, basal total bioactive NOx *versus* FeNO signal in sheep placental tissue as well as liver. FeNOs are only detected in the sheep placenta. *D*, the voltage tracing of the FeNO signal from the sheep placenta. *∗∗ represents p < 0.01*. FeNO, iron nitrosyl complex.

We recently observed evidence of endogenous FeNO in the mouse placenta, indicating that FeNOs might be important under both physiological and pathological conditions (13). We therefore extended this work by assaying for the presence of FeNO in the placentas of three rat strains, the Sprague Dawley, Lewis, and Brown Norway rats, which are commonly used in animal studies of pregnancy or various disease models. Following indirect evidence that the sheep placenta might also produce FeNOs, we assayed for the presence of FeNOs in the sheep placenta. As shown in Figure 2*A*, endogenous FeNOs were detected in the placenta of all three rat species and constituted ∼50% of the bioactive NOx signal detected. Figure 2*B* shows the voltage tracing of the FeNO signal in the rat placenta as detected by the PBS/FeCN assay when six different samples were injected. Endogenous FeNOs were similarly observed in the sheep placenta (Fig. 2*C*) and constituted ∼25% of the bioactive NOx signal detected. Figure 2*D* shows the voltage tracing of the FeNO signal in the sheep placenta as detected by the PBS/FeCN assay when three different samples were injected. We have thus observed endogenous FeNO formation in the placenta of mouse, rat, sheep, and humans, indicating the importance of these species in normal placental physiology.

We recently reported our observation of FeNO formation in the postnuclear fraction of human placental tissue homogenates in the presence of exogenous NO and NOx (nitrite and S-nitrosoglutathione) at concentrations expected in certain maternal inflammation conditions during pregnancy (13). However, similar to the endogenously observed FeNOs in the mouse placenta, the FeNOs thus far observed in the human placenta were too small in magnitude to properly characterize. We therefore extended this work to explants of human placental villous tissue in the hope of detecting higher levels of FeNO to enable further characterization of these NOx species.

**Figure 3:**
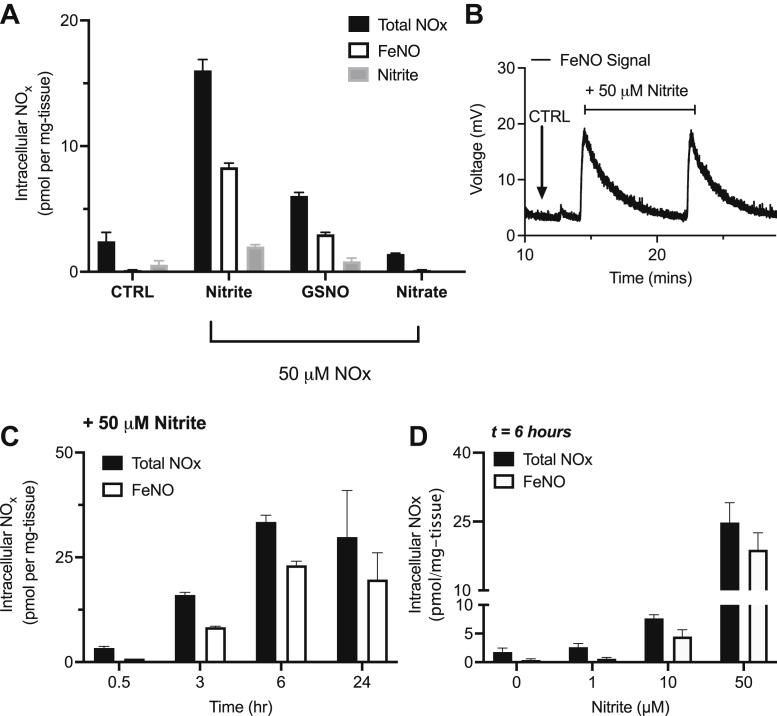
**Detection of intracellular NOx *versus* FeNO in human placental villous tissue explants (n = 3) following exposure to exogenous NOx for 3 h.***A*, formation of FeNO in human placental explants cultured for 3 h in media containing 50 μM of either nitrite, GSNO, or nitrate. Intracellular total bioactive NOx (*black bar* = nitrite, RSNO, and FeNO) is increased in the placental tissue treated with either nitrite or GSNO but not nitrate. At least half of the intracellular NOx signal is in the form of FeNOs (*white bar*). *B*, the voltage tracing of the observed FeNO signal following incubation of human placental explants with media supplemented with 50 μM nitrite. *C*, time dependency of tissue FeNO formation in human placental explants (n = 4) cultured in media supplemented with 50 μM nitrite. Peak FeNO formation is observed at t = 6 h, but FeNO formation is evident starting at 30 min of incubation. *D*, dose dependency of tissue FeNO formation in human placental explants cultured in media supplemented with 1, 10, or 50 μM nitrite for t = 6 h. Peak FeNO formation is observed with 50 μM nitrite, which was used for the rest of the study. FeNO, iron nitrosyl complex; GSNO, S-nitrosoglutathione.

We cultured human placental villous tissue for 3 h with and without media containing 50 μM nitrite, S-nitrosoglutathione, or nitrate, which are all metabolites of NO. Assaying for the NOx within the explant tissue following media removal, tissue washing, and tissue homogenization and centrifugation to collect the postnuclear fraction revealed increased tissue NOx after incubation with either nitrite or S-nitrosoglutathione, and at least half the signal was in the form of FeNOs (Fig. 3*A*). No increases in tissue NOx were observed after incubation with nitrate. The voltage-trace of the observed FeNOs is shown in Figure 3*B*, following injection of supernatant from nitrite-treated explants.

Since nitrite yielded the most FeNOs, we performed all additional experiments of FeNO characterization following treatment of explants with nitrite. We found FeNO formation from nitrite in explants to increase with duration of incubation of the nitrite with explants (Fig. 3*C*) with a peak at 6 h. We also found nitrite-derived FeNO formation to be dose dependent with a peak at 50 μM nitrite (Fig. 3*D*). We therefore incubated placental villous tissue explants for 6 h with 50 μM nitrite for observation and characterization of FeNOs at maximal levels.

**Figure 4:**
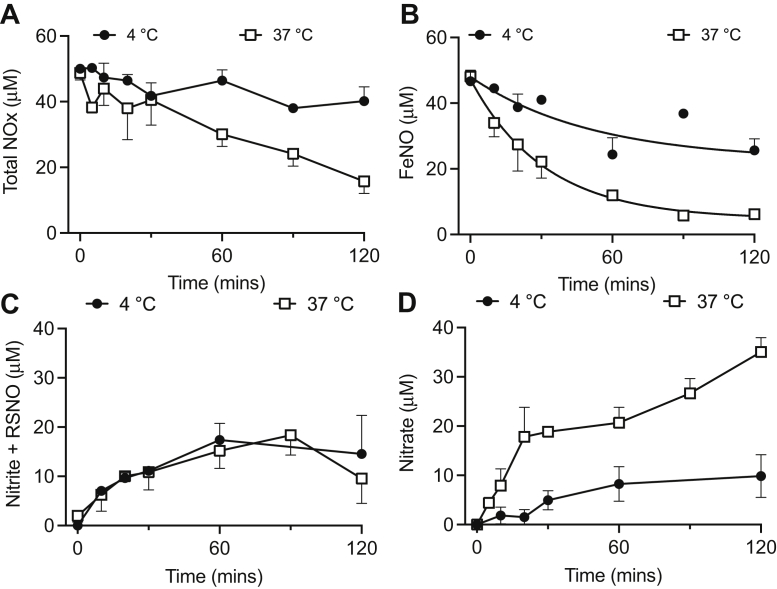
**Characterization of the temporal and thermal stability of placental tissue (n = 4) FeNOs within tissue homogenates.** FeNOs were formed after incubating human placental villous tissue explants with nitrite-supplemented media. Placental villous explant tissue homogenates prepared from human placental villous tissue exposed to 50 μM nitrite for 6 h were placed on ice (4 °C) or in a water bath at 37 °C. Changes in (*A*) total bioactive NOx concentrations (nitrite, RSNO, and FeNO) and (*B*) FeNO concentrations in homogenates were followed for 2 h using ozone-based chemiluminescence. FeNOs decreased exponentially at both 4 °C and 37 °C and were more stable at 4 °C (half-life = 36 min) *versus* 37 °C (half-life = 22 min). The total bioactive NOx signal is generally more stable than the FeNO signal, and the loss of FeNO could be attributed to formation of (*C*) nitrite and RSNO and (*D*) formation of nitrate, which is the predominant metabolite formed. FeNO, iron nitrosyl complex.

We next determined how thermally and kinetically stable the observed FeNOs were under typical assaying conditions. Following nitrite-derived FeNO formation in human placental explants, we processed placental tissue to collect the postnuclear fraction containing FeNOs. We then incubated this FeNO-containing fraction at either 4 or 37 °C for 2 hours within which samples were collected at 0, 5, 15, 30, 60, and 120 min and then assayed for total bioactive NOx by triiodide assay or for FeNO by PBS/FeCN assay. The NOx and FeNO concentrations were relatively more stable at 4 °C than at 37 °C (Fig. 4, *A* and *B*), suggesting that FeNO-containing samples are best assayed at 4 °C. The fall in both NOx and FeNO concentrations was exponential at 37 °C, while at 4 °C, there was essentially no change in NOx (nitrite + RSNO + FeNO) concentration and a linear decrease in FeNO concentration. Thus, at 4 °C, FeNO was predominantly lost to nitrite and RSNO, which are both detectable by triiodide (Fig. 4*C*), while at 37 °C, FeNO was predominantly lost to nitrate (Fig. 4*D*), which is not detectable by triiodide assay without sample pretreatment with NiR enzyme.

**Figure 5:**
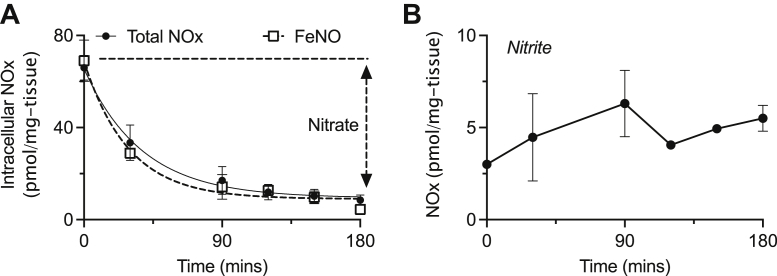
**Characterization of FeNO stability within human placental villous tissue explants.** Placental tissue explants were incubated with media containing 50 μM nitrite for 6 h following which the media was removed and replaced with nitrite-free media. Explants were homogenized at different time points up to 3 h following nitrite removal. *A*, loss of total bioactive NOx (nitrite, RSNO, and FeNO) indicates the kinetics of FeNO degradation following removal of nitrite in human placental explants treated with 50 μM nitrite for 6 h. Both total bioactive NOx and FeNO are lost exponentially, and the magnitude of the bioactive NOx loss is identical to the magnitude of the FeNO loss indicating nitrate formation. *B*, very little nitrite is formed relative to the amount of FeNO that is lost from the placental tissue explants following nitrite removal from the media. FeNO, iron nitrosyl complex.

Since the concentration of FeNOs is most likely a steady-state process between their formation and degradation, we also determined how stable the formed FeNOs were in the tissue following nitrite removal, thus removing the FeNO formation pathway. Following incubation with 50 μM nitrite for 6 h, human villous explants were washed, then blotted to remove any residual nitrite, and then replaced with nitrite-free media. Different explant samples were then collected at time 0, 30, 90, 120, 150, and 180 min from nitrite removal and immediately homogenized and processed for NOx analysis. As shown in Figure 5*A*, both the tissue NOx and FeNO signals were lost exponentially, and the magnitude of the NOx signal was equal to that of the FeNO signal indicating that virtually all the endogenously formed FeNO was lost to nitrate within the placental tissue. The change in FeNO concentrations fit to a one-phase decay curve with a half-life of 26 min for the total NOx and 20 min for FeNO. As shown in Figure 5*B*, there was relatively little change in nitrite in the media from which the explant tissue was removed. Nitrate was thus found to be the product of endogenous FeNO disposal within tissue, a result which is consistent with our recent finding that placental tissue rapidly degraded exogenous DNICs to nitrate, which is essentially inert unless reduced back to nitrite by NiRs (13).

**Figure 6:**
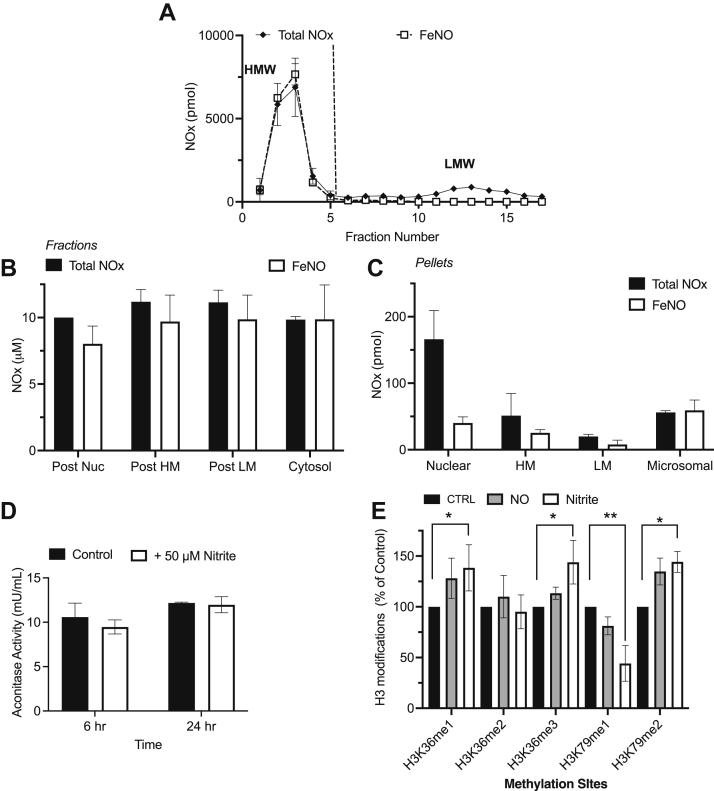
**Molecular weight fractionation and differential centrifugation.***A*, supernatant from homogenates of human placental explants (n = 2) treated with 50 μM nitrite for 6 h was passed through a *G-25 column* to yield *low* molecular weight (LMW) and high molecular weight (HMW) fractions. All the FeNO and total bioactive NOx was located in the HMW fraction indicating that nitrite, a low molecular weight molecule, had been converted into a protein-bound FeNO adduct. Homogenates of human placental explants treated with 50 μM nitrite was separated into different fractions lacking subcellular components through differential centrifugation. The change in FeNO concentrations in the different liquid fractions (*B*) or in the pellets reconstituted in buffer (*C*) was determined by ozone-based chemiluminescence. FeNOs were observed in every subcellular compartment and were more prominent in the heavy mitochondrial and microsomal compartments. *D*, aconitase activity of cultured human placental villous explants (n = 3) and (*E*) change in histone methylation at different methylation sites in human placental villous explants (n = 4), following treatment with 250 μM nitrite or 500 μM DETA, which yields steady-state concentrations of ∼250 nM NO over 24 h. There is no change in aconitase activity following exposure to nitrite. There is a significant increase in the methylation of a number of lysine residues with nitrite but not with NO itself. Statistical analysis was performed with two-way ANOVA followed by a multiple-comparisons test (Fisher’s LSD test). *∗ represents p < 0.05, ∗∗ represents p < 0.01*. DETA, diethylenetriamine; FeNO, iron nitrosyl complex; NO, nitric oxide.

We next used size-exclusion column chromatography to assess the approximate size of the formed FeNO, enabling distinction between low molecular weight FeNOs, likely to be DNICs, or high molecular weight, protein-bound heme- or nonheme-FeNOs. Following passage of supernatant from homogenized nitrite-treated explants, exclusively all the FeNO signal was found in the high-molecular weight fractions (Fig. 6*A*), indicating that the observed FeNO was likely to be protein bound.

We then used differential centrifugation to determine the subcellular location of the potential proteins contributing to the observed FeNO signal. Following differential centrifugation to sequentially remove different subcellular compartments, we observed an FeNO signal in the postnuclear fraction, postheavy mitochondrial fraction, postlight mitochondrial fraction, and cytosolic fractions (Fig. 6*B*), indicating that different proteins localized in different compartments contribute to the FeNO signal. The majority of the FeNO was however localized to the cytosol, since differential centrifugation did not significantly change the FeNO concentration, suggesting the predominance of a cytosolic protein responsible for the observed FeNOs. We then directly assayed for FeNOs within the actual pellets representing different subcellular compartments. The pellets were reconstituted in PBS buffer and then injected into the triiodide and PBS/FeCN assays. The presence of FeNOs was thus observed in the nuclear (Nuc), heavy mitochondrial (HM), light mitochondrial, and microsomal pellets (Fig. 6*C*), supporting the idea that FeNOs are likely formed from multiple proteins in different cellular compartments. FeNOs were most notable in the HM pellet as well as the microsomal pellets, and virtually all the NOx in these compartments was in the form of FeNOs. Nitrite-derived FeNOs are thus protein bound species localized predominantly in the cytosol and also in the nucleus, HM, and microsomal compartments.

We proceeded to check whether the formation of FeNOs in the cytosol and in different subcellular compartments is associated with any change in activity of proteins known to form FeNO. We determined whether nitrite-derived FeNO formation is associated with either loss of aconitase activity or increase in histone methylation, as an index of loss of demethylase activity. As shown in Figure 6*D*, treatment of placental explants with 50 μM nitrite for 6 or 24 h did not result in any loss of aconitase activity. Aconitase DNIC formation is therefore likely not a major contributor to the observed FeNOs, a result which we later confirmed by electron paramagnetic resonance (EPR). As shown in Figure 6*E*, incubation of placental explants with nitrite was, however, associated with a significant increase in histone methylation of at least three lysine residues (H3K36me1, H3K36me3, and H3K79me2), and a decrease in histone methylation at H3K79me1. No change in histone methylation was observed at lysine residue H3K36me2 as well as ten other residues: H3K4me1, H3K4me2, H3K4me3, H3K9me1, H3K9me2, H3K9me3, H3K27me1, H3K27me2, H3K27me3, H3K36me2, and H3K79me3 (data not shown). The change in histone methylation in at least four lysine residues of nitrite-treated explants relative to controls suggests that JMJC domain-containing demethylases form part of the observed FeNOs. Formation of FeNO in the iron-containing catalytic subunit of the demethylases could result in the observed increase in methylation at the specific sites, due to a loss of demethylase activity.

**Figure 7:**
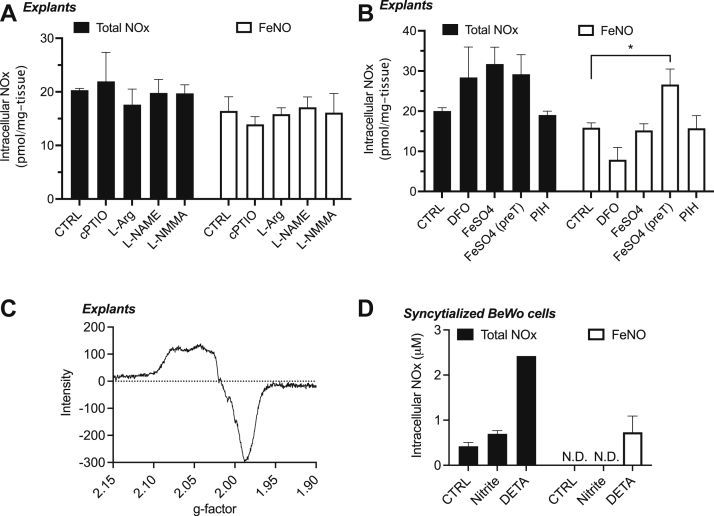
**EPR and pharmacological characterization of FeNO formation from nitrite in human placental villous tissue explants (n = 3)**. Total bioactive NOx *versus* FeNO formation was determined in placental explants cotreated with 50 μM nitrite and different pharmacological agents targeting, (*A*) NO-related pathways, and (*B*) Iron-related pathways. Placental FeNO formation from nitrite does not require NOS enzymes nor the intermediacy of NO. Placental FeNO formation from nitrite is augmented by iron pretreatment before nitrite incubation, but not iron cotreatment with nitrite incubation. *C*, EPR characterization of FeNOs reveals the triplet signal at g = 2.00, which is characteristic of heme-NO. *D*, nitrite-derived FeNO formation is not found in cultured BeWo cells (n = 3), before or after syncytialization, even though nitrite increases the total intracellular NOx within the cultured cells. *∗ represents p < 0.05*. EPR, electron paramagnetic resonance; FeNO, iron nitrosyl complex; NO, nitric oxide; NOS, nitric oxide synthase.

To further explore the mechanism by which nitrite, a small molecule with limited reactivity, is converted to an NO moiety bound to an iron center in a protein, we utilized different pharmacological agents targeting both the NO and iron metabolism pathways, in the hope of modulating the amount of FeNOs formed from nitrite. Incubating placental explants with nitrite along with cPTIO, a NO scavenger, did not attenuate nitrite-derived FeNO formation (Fig. 7*A*), suggesting that the formation of FeNOs from nitrite does not involve the production of free NO gas as an intermediate. The nitrite-derived FeNO levels were also neither increased by incubation with L-arginine, the substrate for NOS enzymes, nor decreased by incubation with L-NAME and L-NMMA (Fig. 7*A*), which are competitive inhibitors of NOS enzymes. Thus, the observed production of the FeNOs most likely does not involve the enzymatic generation of NO from NOS enzymes.

In addition to exploring the potential source of NO in the nitrite-derived FeNOs, we also explored the involvement and potential source of iron in the FeNO formation. FeNOs are typically complexes of NO with a heme- or nonheme iron center of proteins or involve the complexation of chelatable iron with NO to form low molecular weight DNICs. We therefore cotreated placental explants with both nitrite and 100 μM of either the iron chelators deferoxamine (DFO) and pyridoxal isonicotinoyl hydrazone (PIH) or with iron sulfate as a source of supplemental iron. As shown in Figure 7*B*, chelation of free iron by both PIH and DFO did not result in a significant decrease of FeNO formation from nitrite. Treatment with DFO tended to decrease FeNO production, but this experiment was confounded by the previously unknown property of DFO whereby it also releases NO under our assay conditions regardless of the presence of placental tissue. We have reported this property (24) and mathematically accounted for the NO released from DFO in our reported results. The results of iron chelation by DFO and PIH suggest that FeNO production from NO_2_^-^ might not be immediately dependent on chelatable iron and thus are not likely to be low molecular weight DNICs, also consistent with our size-exclusion chromatography results.

Cotreatment of placental explants with nitrite and 100 μM iron for 6 h also did not yield any significant increase in FeNOs (Fig. 7*B*), again suggesting FeNO formation was not dependent on chelatable iron. Pretreatment of the explants with the same concentration of iron for 12 to 24 h, thus allowing for the possible incorporation of iron into proteins, followed by treatment with nitrite for 6 h, resulted in a significant ∼30% increase in measured FeNOs. This experiment supports the suggestion from the size-exclusion experiments that FeNO formation requires protein-bound iron. This iron could, however, be bound to heme proteins resulting in heme-NO or to iron sulfur cluster proteins resulting in high molecular weight DNICs. As shown in Figure 7*C*, following EPR studies to detect either paramagnetic mononuclear DNICs or heme-NO, the abundant FeNO signal was in the form of heme-NO with the characteristic triplet peak evident at g = 2.00. No DNIC signal was detected by EPR, and exposure of placental explants to nitrite did not alter mitochondrial aconitase activity (Fig. 6*D*), which is known to decrease in conditions where the aconitase iron sulfur cluster is converted to a DNIC following binding to NO. The lack of change in aconitase activity is therefore consistent with the EPR result, in which heme-NO, and not DNICs, seem to be the predominant FeNO formed in placental explants following exposure to nitrite.

Since heme-NO seems to be the predominant FeNO formed in placental villous tissue following nitrite exposure and since the predominant part of FeNO signal was located in the cytosol (Fig. 6*B*), it is possible that fetal hemoglobin nitrosyl complex forms a significant part of the pool of the observed FeNOs. A small amount of fetal hemoglobin remains trapped within the villous explant tissue following tissue processing before culture and thus is a major source of the heme present in the cytosol following tissue homogenization. We recently observed that fetal hemoglobin forms iron nitrosyl hemoglobin (HbNO) in the presence of DFO and does so more readily than maternal hemoglobin (24). Fetal hemoglobin has also been shown to more efficiently reduce nitrite compared to adult hemoglobin, resulting in the formation of HbNO, following reaction of fetal/adult deoxyhemoglobin with nitrite (25). It is therefore highly likely that most of the observed FeNOs are in the form of fetal HbNO and/or that the observed FeNOs require the participation of fetal hemoglobin.

We therefore eliminated the contribution of fetal hemoglobin by incubating nitrite with BeWo cells, either in mononuclear (cytotrophoblast) or multinucleated (syncytiotrophoblast) form, instead of tissue explants. In addition, experiments were carried out at 21% O_2_ to test for the direct production of FeNOs from nitrite while minimizing confounding FeNO production from the hypoxic reduction of nitrite to free NO. As shown in Figure 7*D*, incubation of syncytialized BeWo cells with either nitrite or diethylenetriamine (DETA), a nitric oxide donor, both resulted in an increase in intracellular NOx as measured by triiodide assay. Nitrite however did not yield any detectable FeNOs within the syncytialized BeWo cells as assayed by the PBS/FeCN assay. Similar results were observed in BeWo cells before induction of syncytialization (data not shown). The absence of FeNOs in BeWo cells cultured with nitrite, in contrast to the presence of these FeNOs in human placental villous tissue, is consistent with the involvement of fetal hemoglobin in placental tissue FeNO formation following nitrite exposure. Fetal HbNO also likely constitutes a significant portion of the observed FeNO signal, although our results with differential centrifugation suggest that FeNOs are also present in other subcellular compartments especially the HM and microsomal cellular compartments (Fig. 6*C*).

## Discussion

Nitric oxide plays an important role in normal pregnancy as well as pregnancy complications such as fetal growth restriction, preeclampsia, and gestational diabetes. The changes in plasma NO metabolites in these conditions are however not as clear since they have been shown to increase, decrease, or remain unchanged relative to levels in normal pregnancy (11). We recently reported that levels of NOx were increased in placental tissue from pregnancies associated with fetal growth restriction, with or without preeclampsia (13). Since maternal inflammation is associated with increases in placental NO synthesis, we determined the levels of NO metabolites at the placental tissue level, instead of using plasma levels as a proxy, since the latter are more markedly affected by dietary changes in nitrite and nitrate. We have thus found the levels of both nitrate and NOx (sum of nitrite, nitrosothiols, and FeNOs) to be increased in placental tissue from pregnancies associated with preeclampsia, independent of histologic villitis, compared to placental tissue from normal pregnancies. This result adds to the studies that suggest an important role for NO in preeclampsia, although it is not clear whether increased levels of NO play an adaptive or pathologic role. A deeper understanding of NO handling in the placenta is needed in order to make progress on this front.

FeNOs have the potential to act as either sinks or stores of NO, thus modulating the amount of free NO able to perform known biological functions. FeNOs also represent a mode of NO-mediated posttranslational modification of heme or nonheme iron-containing proteins, as their formation could result in the alteration of protein activity. We have previously detected FeNOs in blood from the fetal sheep umbilical vein, but not in maternal or umbilical arterial blood (26), suggesting that the sheep placenta produces FeNOs. We have also demonstrated the existence of FeNOs in the healthy mouse placenta (13). In the present work, we confirm the existence of FeNOs in sheep placental tissue, as well as in the placenta of Sprague Dawley, Lewis, and Brown Norway rat strains. We also note that placentas from the Brown Norway rats, which serve as a model of preeclampsia and placental insufficiency (27, 28), have higher total NOx and FeNO than placentas from either the Sprague Dawley or the Lewis rats. Together, these results point toward a physiological function of FeNOs in the placenta. It is possible that the placental FeNO is derived from nitrite (or nitrate following conversion to nitrite) circulating in the bloodstream, as it can diffuse freely across the placenta (29). Most of this nitrite is thought to be derived from eNOS, but it can also be derived from dietary intake of nitrite and nitrate (30). It is also possible that the FeNO production is derived from nitrite that is formed within the placenta due to oxidation of NO produced locally by constitutively expressed eNOS.

Although FeNOs were not directly detected in the normal human placenta at term, they were readily formed in homogenates following exposure to NO and even nitrite (13), which is less reactive than NO and requires reduction en route to FeNO formation (31). However, the FeNOs that we have observed thus far, in either the animal or human placenta following exposure to NO or nitrite, were too low in concentration to permit further characterization of their nature and stability. Using human placental villous explants cultured in the presence of various NOx species, we have demonstrated that nitrite treatment results in the formation of FeNOs in placental tissue at concentrations sufficient to enable further characterization by ozone-based chemiluminescence, EPR, and different protein activity assays.

Using ozone-based chemiluminescence with either triiodide or PBS/FeCN assays, we have demonstrated that placental FeNOs have a relatively short half-life of ∼20 min in both native tissue before homogenization and in tissue homogenates at 37 °C. The half-life improves to 36 min in nitrite-treated placental villous explants homogenates at 4 °C, therefore, it is important to rapidly perform all placental tissue collection, processing, and assay measurements on ice so as to limit FeNO degradation and enable better detection of these labile species. We also observed that FeNOs are preferentially degraded to nitrate in both native placental tissue and tissue homogenates at 37 °C, but are degraded mainly to nitrite at 4 °C. This result is consistent with our observation that exogenous FeNOs in the form of glutathione-liganded BDNIC were rapidly (half-life of 6 min) degraded to nitrate by placental homogenates (13). Since the endogenous placental tissue FeNOs were formed from nitrite and rapidly degrade to nitrate under nonsteady state conditions, the human placenta can thus be seen as oxidizing nitrite to the relatively more inert nitrate through an FeNO intermediate. Thus, FeNOs can serve as a sink of NO bioactivity, and their formation, though at high levels, might signal an effective lack of NO availability.

Alternatively, FeNOs could also be acting to transiently modulate placental function. As demonstrated by chemiluminescence, nitrite-derived FeNOs were found in multiple subcellular compartments, such as the nucleus, and notably the heavy mitochondria and microsomes, where virtually all NOx was in the form of FeNO. The mitochondria houses several heme-iron–containing proteins such as mitochondrial aconitase involved in the Krebs cycle, as well as cytochrome complex and complexes I, II, III, and IV, all of which are involved in the electron transport chain (32). Mitochondrial aconitase is known to form DNICs following exposure to NO in both tissue and in purified aconitase samples. This DNIC formation is often associated with a decrease in aconitase activity (19, 33, 34). We did not detect any DNIC signal by EPR in nitrite-treated explants, however, and an assay for aconitase activity did not show any difference between nitrite-treated explants and controls. DNIC formation is therefore not likely to be a major contributor to the observed FeNOs, a conclusion also supported by the EPR experiment which measured heme-NO but not DNICs.

Another emerging mode of NO signaling that is more recently recognized is its role in epigenetic regulation of gene expression. The mechanism may be via posttranslational protein modifications, such as S-nitrosylation and nitration resulting in altered function of transcription factors (35, 36) or direct modification of histone protein structure (37). In addition, and particularly relevant to the current observation of FeNO formation, NO has also been shown to modify global histone methylation by inhibiting Jumonji C domain-containing demethylases through formation of an FeNO complex in the catalytic core of the demethylase enzyme (22, 38, 39). Our observation that nitrite-derived FeNOs were associated with an increase in histone methylation at a number of lysine residues is in support of a role for FeNOs in epigenetic regulation. However, future studies are needed to identify the exact proteins on which FeNOs form in the placenta.

While we observed FeNOs being formed from nitrite within placental tissue, we are yet to fully understand the exact mechanism of this reaction. So far, we understand that nitrite, as low as 1 μM and as early as 30 min, yields an NO moiety that binds to ferrous iron, to yield an FeNO. Oxidation of this FeNO by FeCN releases NO gas that is detected by the NO analyzer, yielding a signal that is not present with placental explants alone, or with nitrite alone. Through size-exclusion chromatography, we understand that the ferrous iron with which nitrite reacts is part of a protein, and not just free iron. We also understand through EPR that the observed FeNO is predominantly in the form of heme-NO and not DNIC, and through differential centrifugation, we understand that most of the heme-NO was located in the cytosol, although some signal was also located in the nuclear, HM, and microsomal pellets. We also observed that the FeNO signal was increased by pretreatment but not cotreatment with ferrous iron sulfate, once again supporting the idea that the FeNO is protein bound, requiring some time for the iron to be integrated into proteins, instead of being a complex with low molecular weight, chelatable iron. This idea is supported by cell-culture experiments using a hemoglobin-free system of cultured BeWo cells, before or after syncytialization, where no FeNOs are detected following exposure to nitrite. Thus, most of the FeNO that we observe is most likely to be fetal iron HbNO, and the presence of trace amounts of hemoglobin might be a prerequisite for FeNO formation within the placenta (40).

The abundance of the cytosol FeNO signal, and its identification as heme-NO, coupled with the knowledge that some fetal hemoglobin remains trapped within villous tissue following tissue processing points toward fetal hemoglobin being the predominant heme-protein resulting in the observed FeNO signal. This idea is supported by cell-culture experiments using a hemoglobin-free system of isolated placental cytotrophoblast and syncytiotrophoblast cells, where no FeNOs are detected following exposure to nitrite at 21% O_2_. Thus, most of the FeNO that we observe is likely to be fetal iron HbNO, and the presence of trace amounts of hemoglobin might be a prerequisite for FeNO formation within the placenta.

Fetal deoxyhemoglobin is known to reduce nitrite to NO gas with much better efficiency than maternal hemoglobin, resulting in the concomitant formation of HbNO especially under hypoxic conditions (25, 41). It is therefore possible that FeNO formation is due to nitrite reduction to NO by fetal deoxyhemoglobin trapped within the villous explants. At odds with this possibility, we note that FeNO production from nitrite was observed under room-air oxygenated conditions and at physiological pH in both the current experiments and in our previous work with oxygenated placental tissue homogenates (13). As such, fetal hemoglobin would be expected to be nearly 100% oxygenated and would thus not only be unable to reduce nitrite to NO but would also scavenge NO at nearly diffusion-limited rates to form nitrate and methemoglobin (42). Furthermore, we found that the presence of the NO scavenger CPTIO had no effect on FeNO production from nitrite. Our results therefore suggest that the formation of FeNOs from nitrite does not require the production of free NO gas as an intermediate. Although we do not know the precise NO-independent mechanism of heme-NO formation, we similarly observed the formation of fetal HbNO from DFO through a mechanism that was NO independent (24). Bryan *et al*. also observed the nitrite-derived formation of FeNOs in multiple organs of the normoxic male Wistar rat and similarly observed the FeNO formation and other bioactivity of nitrite to be NO independent (24, 43). This accumulation of evidence therefore calls for increased study into the reaction by which nitrite forms FeNOs without the intermediacy of free NO, which might lead to a better understanding of nitrite’s NO-independent biological activity.

In summary, we hereby report the efficient formation of FeNOs from nitrite in explants from human placental villous tissue, as well as the endogenous formation of these complexes in the placenta of healthy sheep and three different rat strains. Although nitrite is a small molecule that was previously considered to be an inert product of NO metabolism, the efficient formation of FeNOs from nitrite in the human placenta hints toward the importance of both nitrite and FeNOs in placental physiology or pathology. Future studies will explore the exact mechanism through which FeNOs form from nitrite and the specific proteins on which these complexes form.

## Experimental procedures

### Ethical approval

Human placentas were collected at two different sites: Loma Linda University Medical Center for studies of the metabolism of nitrite in human placental villous explants and the University of California San Diego for measurement of basal NOx levels in placental homogenates from normal or preeclamptic pregnancies. Collection of human placentas for the study of nitrite metabolism was approved by the Loma Linda University Institutional Review Board (IRB #5180266). Collection of human placentas for measuring the basal NOx levels and species in age-matched normal *versus* preeclampsia placentas was carried out through the UC San Diego Perinatal Biorepository, under a protocol approved by the University of California San Diego Institutional Review Board (IRB #181917), and all mothers provided their written, informed consent for the use of their placentas.

All animal procedures and protocols were approved by the Institutional Animal Care and Use Committee of Loma Linda University and followed the guidelines of the National Institutes of Health Guide for the Care and Use of Laboratory Animals (44).

All studies in this work abide by the Declaration of Helsinki principles.

### Tissue collection

#### Rat placenta

Three-month-old outbred Sprague-Dawley rats, Brown Norway inbred rats, and Lewis inbred rats from barrier H49 were obtained from Charles River Laboratories. All rats were housed at the Loma Linda University Animal Research Facility under conditions of 14 h of fluorescent light, 10 h of darkness, ambient temperature of 20 °C, and relative humidity of 30 to 60%.

Rats were bred by overnight monogamous pairing of a virgin female with a male breeder. The following day was considered day 0 of pregnancy, and pregnancy was confirmed by examining vaginal plugs on day 0. To study endogenous levels of placental FeNOs, female rats were euthanized by heart/lung bloc removal while under isoflurane anesthesia (4%) at various stages following pregnancy confirmation. Sprague-Dawley rats were sacrificed at gestational age P18, and both the Lewis and Brown Norway rats were sacrificed at gestational age P17. Before heart removal, blood was quickly collected, directly from the heart, using EDTA blood tubes containing 0.01 mM p-hydroxyl-mercury benzoate, 1.5 mM o-phenanthroline, 0.01 mM PMSF, and 0.05 mM pepstatin A, followed by separation of plasma at 3000*g* 5 min at 4 °C and storage at −80 °C. Placental as well as other tissues were harvested, weighed, snap frozen in liquid nitrogen, and stored at −80 °C for later analysis.

#### Sheep placenta

Near-term– (137–140 days of gestation, term = 150 days) mixed western breed pregnant ewes were obtained from Nebeker Ranch. After an overnight fast, blood was collected from the jugular vein of unanaesthetized adult ewes into heparinized syringes using an 18 gauge needle and centrifuged at 1000*g* for 15 min within 30 min of blood collection. The ewe's anesthesia was then induced with a bolus of ketamine (10 mg kg^−1^) and midazolam (0.5 mg· kg^−1^) followed by intubation and ventilation with 3% isoflurane. The placenta was then accessed via a midline incision in the abdomen for collection of the cotyledons. After further collection of fetal and maternal tissues for other projects, the ewe and fetus were given a lethal intravenous dose of Euthasol (Virbac Corporation). Placental cotyledons were placed in a tube with PBS buffer, which was then placed on ice, and transported to the cell culture hood for processing to make villous explants within 30 min of collection. Villous explants were prepared from the freshly obtained cotyledons as described for the human placenta.

### Human placenta: measurement of basal NOx levels in preeclampsia

Placentas for preeclampsia were obtained from patients with either superimposed or severe preeclampsia, as defined by the American College of Obstetricians and Gynecologists (45). Villitis was defined as the presence of a mixed infiltrate of lymphocytes and macrophages within chorionic villi and ranged from low- to high-grade based on the criteria published by the Amsterdam Workshop (46).

Villous tissue, sampled from the intervillous space between the basal and chorionic plates, was collected from placentas between 34 and 41 weeks of gestation from: (i) normotensive pregnancies without preeclampsia, fetal growth restriction, or villitis, or (ii) preeclamptic pregnancies without both fetal growth restriction and villitis. For all placentas, tissue was snap-frozen in liquid nitrogen within 90 min of delivery and stored at −80 °C until homogenization for NOx analysis. Collection of placentas was made without selection for either mode of delivery (vaginal *versus* Caesarian section) or labor status (laboring *versus* nonlaboring). Maternal and neonatal demographic information are summarized in Table S1.

### Placental explant culture

Term placentas (39 weeks) were collected from patients without any pregnancy complications, within 30 to 60 min following vaginal or Caesarian section birth. Following the weighing of the placenta without umbilical cord and separation from the amniotic chorionic membrane, villous tissue was randomly sampled from multiple placental cotyledons by avoiding the chorionic plate and trimming off the decidual layer. At least, six villous samples (∼100 mg) were snap frozen for later RNA isolation. The remaining villous tissue from different placental sections was washed in PBS (pH = 7.4) to remove excess blood, then dissected into 5 to 15 mg villous explants for tissue culture in a 10 × 15 mm culture plate. Three individual explants were randomly selected from the culture plate and placed in each well of a 12-well plate with 1.5 ml of complete Iscove’s modified Dulbecco’s medium with 10% fetal bovine serum, 1% each of L-glutamine and penicillin/streptomycin mixture. All culture experiments were performed in an incubator at 37 °C, 5% CO_2_ and 21% O_2_. Although 21% O_2_ likely results in oxygen concentrations at the tissue-buffer interface that are higher than those of placental tissue *in vivo*, this concentration was chosen to minimize the possibility that O_2_ levels for cells within the tissue explants were hypoxic or anoxic (47, 48).

To determine the acute effect of nitrite on human placental function, human placental villous explants were cultured in Iscove’s modified Dulbecco’s medium with and without 1 to 50 μM nitrite and placed in a humidified incubator for 30 min, 3, 6, or 24 h. Three wells were used per experimental condition, and following incubation, the villous tissue was washed twice with PBS (pH = 7.4) followed by blotting on paper towel to make sure all nitrite was removed. Villous tissue from all three replicates per experimental group were then combined and snap frozen for later NOx analysis and RNA isolation.

### Tissue processing

#### Basal NOx metabolite levels: normal *versus* preeclampsia placentas

The homogenization of villous tissue from normal and preeclampsia-associated placentas was performed in cold PBS at pH 7.4 without calcium or magnesium (Gibco, Life Technologies). Each 100 mg of villous tissue was placed in 1 ml of buffer, followed by homogenization on ice (TissueRuptor, Qiagen). The homogenate was centrifuged at 1000*g* for 15 min at 4 °C to remove tissue debris, and part of the supernatant was collected and placed on ice for immediate NOx analysis, whereas another part was snap-frozen in liquid nitrogen and stored at −80 °C for NOx analysis within 1 day of tissue homogenization.

#### Placental explants NOx following treatment with NOx

Following incubation in media-containing different NOx species, placental explants were washed twice with cold PBS and blotted on dry paper. The explants were then added to PBS buffer (1 ml PBS per 1 mg tissue), then homogenized on ice (TissueRuptor, Qiagen), and the homogenate was centrifuged at 1000*g* for 15 min at 4 °C to remove tissue debris. The supernatant was then immediately assayed for NOx and FeNO by ozone-based chemiluminescence with either triiodide or ferricyanide-based assays as fully described below.

#### Preparation of subcellular fractions by differential centrifugation

To investigate FeNO formation in cellular compartments, homogenates prepared from nitrite-treated placental villous tissue explants were fractionated by differential centrifugation (49). In brief, nitrite-treated villous tissue was washed with and homogenized in pH 7.4 PBS (8 μl per mg-tissue), followed by centrifugation for 15 min at 1000*g* to remove tissue debris and nuclei. The pellet (nuclei) was resuspended in pH 7.4 PBS. The postnuclear supernatant was centrifuged at 3,000*g* for 10 min and the pellet corresponding to the HM fraction was resuspended in pH 7.4 PBS buffer. For the light mitochondrial fraction preparation, the supernatant was further centrifuged at 16,000*g* for 10 min and the pellet was resuspended in pH 7.4 PBS buffer. The postlight-mitochondrial supernatant was centrifuged at 100,000*g* for 45 min to collect the cytosol fraction, and the microsomal pellet was collected and resuspended in pH 7.4 PBS buffer. NOx content for each fraction was measured separately by ozone-based chemiluminescence.

#### Ozone-based chemiluminescence

NO was measured by an ozone-based chemiluminescence analyser (NOA 280i; Sievers). Different purge vessel reagents were used to determine the concentrations of specific types of NOx species formed (or lost) following recently published protocols that allow for discrimination between nitrate, nitrite, nitrosothiols, and NO bound to iron (FeNO) in the form of DNICs and heme nitrosyls (hemeNO) (23). In brief, triiodide reagent was used (assay referred to as I3^-^) to determine the presence of all NOx species, comprised mainly of nitrite, nitrosothiols, N-nitrosyls, and FeNO, but not nitrate. We hereafter refer to the sum of all NOx species (except nitrate) as total NOx. Nitrate concentration was determined by incubating the sample for 45 min at 37 °C with bacterial NiR (Sigma), flavin adenine dinucleotide (Sigma), and NADPH (Sigma), followed by injection in triiodide reagent (NiR/I3), as described previously (Kanady *et al*. 2012). Then, 1 ml of 0.8 M potassium ferricyanide added to 4 ml of PBS buffer at pH 7.4 (assay referred to as PBS/FeCN) was used to selectively measure FeNO without nitrite and nitrosothiol interference, as previously described (23). Neither nitrite nor nitrosoglutathione yield a signal in PBS/FeCN. All assays with PBS included 100 μl of 1-octanol as an antifoam agent when biological samples were being measured.

NO-release was monitored in real time using proprietary software (Liquid version v3.21, Sievers), which was also used to report the area-under-the-curve for each NO-release profile and for calibration of the reagents and quantification of the NO_x_ species concentrations. The NO-release peaks were exported to Prism 9.0 software (Graphpad Software, Inc) for better display.

### Electron paramagnetic resonance

Identification of iron nitrosyl species as either DNICs or heme FeNOs was performed by EPR. EPR spectra of homogenates prepared from control or nitrite-treated placental explant samples were measured at 150 K using a Bruker X-Band EMX Plus EPR spectrometer with a cavity of high sensitivity as previously described (50). The EPR was set to a microwave power of 20 mW, microwave frequency of 9.34 GHz, attenuator of 10 dB, modulation amplitude of 1 G, modulation frequency of 100 kHz, time constant of 20.48 msec, conversion time of 81.92 msec, harmonic of 1, and number of scans of 2.

### Aconitase activity assay

Aconitase enzyme activity was determined in control *versus* nitrite-treated placental villous tissue explants using the Aconitase Activity Colorimetric Assay Kit from BioVision (Biovision Inc). Aconitase activity was measured by the amount of isocitrate, which is converted from citrate by the catalysis of aconitase.

### Histone 3 methylation assay

To assess how nitrite treatment influenced posttranslational modifications of histone H3 in placental villous tissue, the EpiQuik Histone H3 Modification Multiplex Assay Kit was used (Epigentek). Histones were extracted from the placental explants previously collected using the EpiQuik Total Histone Extraction Kit (Epigentek) and used for quantification of methylation status. All assays were carried out following the manufacturer's instructions.

### Cell culture

BeWO cells, a trophoblast cell line (ATCC, CCL-98), were cultured according to the manufacturer's protocol. Briefly, BeWo cells were cultured in Ham's F12 (Kaighn’s) growth medium (Gibco) enriched with 10% fetal bovine serum (Gibco) and antibiotics (100 U/ml penicillin/Streptomycin; both from Gibco). Cells were routinely maintained in T75 flasks (Southern Labware) at pH 7.4 under 21% O_2_, 5% CO_2_, and 95% humidity and 37 °C. Cells were passaged when reaching confluency of 70 to 80%. To investigate FeNO formation following incubation with nitrite, BeWo cells were allowed to reach 50 to 60% confluency at which point fresh media containing treatments of either 500 μM DETA (∼250 nM NO over 24 h, Cayman) or 250 μM NaNO_2_ (Sigma) was given. Cells were incubated with treatment for 24 h. To study FeNO formation in cultured syncytiotrophoblasts, BeWO cells were allowed to reach 50 to 60% confluency and induced to differentiate with 50 μM forskolin (Cayman Chemical) for 48 h. After 48 h, forskolin-containing media was removed and fresh forskolin-free media was added along with respective treatments for another 24 h. The NO group received 500 μM DETA-NONOate (Cayman Chemical) and the nitrite group received 250 μM NaNO (Sigma). At the end of the 24 h, cells were collected from all the flasks and immediately processed. Cells were washed with 1× Dulbecco's phosphate-buffered saline (Gibco) three times, scraped off the culture flasks, and diluted in 500 μl of 1× Dulbecco's phosphate-buffered saline. Cells were subjected to three freeze-thaw cycles to lyse them and then injected into the purge vessel to quantify for NOx as previously described.

### Statistical analysis

The results are expressed as the mean ± SEM. *p* < 0.05 was considered statistically significant. Unless otherwise specified, paired comparisons were performed with Mann-Whitney test, and grouped analyses were performed with Ordinary 1-way ANOVA with Tukey's multiple comparisons test. All analyses were carried out using Prism, version 8.0.

## Data availability

All data are contained in the article. Raw data are available on request by e-mailing abblood@llu.edu.

## Supporting information

This article contains supporting information: Table S1 contains the maternal and neonatal demographic information associated with the placentas used in this study.

## Conflict of interest

The authors declare that they have no conflicts of interest with the contents of this article.
