# Supplementary material for: Iron nitrosyl complexes are formed from nitrite in the human placenta
Source: J Biol Chem. 2022 May 26;298(7):102078. doi: 10.1016/j.jbc.2022.102078 (PMC9257420; doi:10.1016/j.jbc.2022.102078)
Supplement: Supporting information [file mmc1.docx]

**Supplementary Table 1.**

|  | Normal | PE (+/- villitis) | PE (- villitis) | PE (+ villitis) |
| --- | --- | --- | --- | --- |
| n | 20 | 18 | 11 | 7 |
| Male | 10 | 9 | 6 | 3 |
| Female | 10 | 9 | 5 | 4 |
| Gestational age (weeks) | 39.2 ± 1.2 | 37.2 ± 1.3 ‡ | 37.0 ± 1.3 ‡ | 37.5 ± 1.4 † |
| Centile birthweight | 46.8 ± 21.7 | 47.3 ± 27.3 | 50.3 ± 26.7 | 42.9 ± 29.8 |
| BMI | 25.5 ± 7.5 | 32.7 ± 8.3 ‡ | 31.5 ± 9.3 | 34.4 ± 6.7 † |

Data are presented as the mean ± SD.

† *P* < 0.05 vs. normal, ‡ P < 0.01 vs. normal

PE, preeclampsia; BMI, body mass index
